# Tight control of fluid balance may reduce incidence of intra-abdominal hypertension in patients after major abdominal surgery and trauma: a pilot study

**DOI:** 10.1186/cc10847

**Published:** 2012-03-20

**Authors:** P Szturz, J Maca, J Neiser, J Jahoda, R Kula, JT Tichy

**Affiliations:** 1 Faculty Hospital Ostrava, Czech Republic; 2 Yeovil District Hospital, Yeovil, UK

## Introduction

Massive fluid resuscitation followed by hypoperfusion abnormalities in the ICU is a risk factor for development of intra-abdominal hypertension (IAH). The aim of our study was to determine what influence would have the control of daily fluid balance on the incidence of IAH in patients after extensive abdominal surgery or after abdominal trauma.

## Methods

A prospective observational study included a total of 82 adult patients (age: 59 ± 10 years, APACHE II score at admission: 18 ± 11, predicted mortality according to APACHE II score at admission: 34%, observed in-hospital mortality: 20%), number of patients with intra-abdominal pressure (IAP) above 12 at admission: 23 (28%), admitted to a single ICU with the diagnosis of abdominal trauma (*n *= 22) or after abdominal surgery (*n *= 60). During first 7 days the fluid intake and balance was monitored and corrected at 6-hour intervals not to exceed 1,500 ml positivity over 24 hours (oncodiuretic therapy was administered - repeated boluses of starch solutions and/or albumin followed by furosemide). IAP was measured from admission twice daily (standardized measurement by instillation of 25 ml normal saline into the bladder). A sustained elevation of the IAP above 12 mmHg in two consecutive measurements was considered as IAH.

## Results

**Figure 1:**
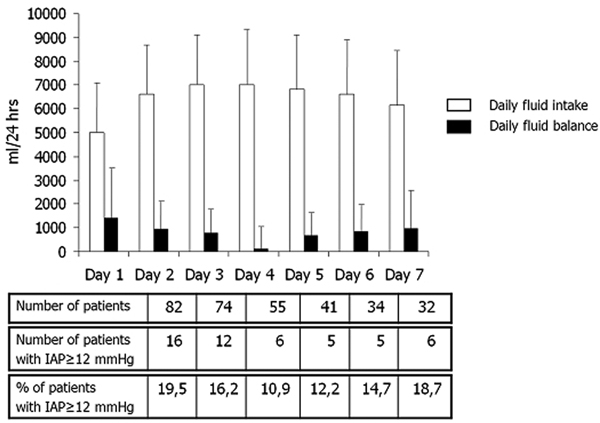
**Fluid intake, fluid balance and incidence of IAH**.

The incidence of IAH in relation to daily fluid intake and daily fluid balance is shown in Figure 1.

## Conclusion

The incidence of IAH in patients after abdominal surgery or abdominal trauma may exceed the value of 40%, especially in situations associated with massive fluid resuscitation [1]. We have identified a close relationship of the daily dynamics of changes in IAP and fluid balance. When we maintained the daily fluid balance not to exceed the positivity of 1,500 ml/24 hours, the incidence of IAH in our study dropped from 28% to less than 20%, despite high daily fluid intake (about 5,000 to 8,000 ml/day). Tight control of the fluid balance seems an effective method to prevent the development of IAH.
